# Increasing Physical Activity in Mothers Using Video Exercise Groups and Exercise Mobile Apps: Randomized Controlled Trial

**DOI:** 10.2196/jmir.9310

**Published:** 2018-05-18

**Authors:** Maya Nina Mascarenhas, June Maylin Chan, Eric Vittinghoff, Erin Lynn Van Blarigan, Frederick Hecht

**Affiliations:** ^1^ Osher Center for Integrative Medicine University of California, San Francisco San Francisco, CA United States; ^2^ Department of Epidemiology & Biostatistics University of California, San Francisco San Francisco, CA United States; ^3^ Department of Urology University of California, San Francisco San Francisco, CA United States

**Keywords:** mobile applications, videoconferencing, Internet, health promotion, exercise, social support, mothers, randomized controlled trial

## Abstract

**Background:**

Women significantly decrease their activity levels in the transition to motherhood. Digital health technologies are low cost, scalable, and can provide an effective delivery mechanism for behavior change. This is the first study that examines the use of videoconferencing and mobile apps to create exercise groups for mothers.

**Objective:**

The aim of the study was to test the feasibility, acceptability, and effectiveness of an individually adaptive and socially supportive physical activity intervention incorporating videoconferencing and mobile apps for mothers.

**Methods:**

The Moms Online Video Exercise Study was an 8-week, 2-armed, Web-based randomized trial comparing the effectiveness of a group exercise intervention with a waitlist control. Healthy mothers with at least 1 child under the age of 12 years were recruited through Facebook and email listservs. Intervention participants joined exercise groups using videoconferencing (Google Hangouts) every morning on weekdays and exercised together in real time, guided by exercise mobile apps (eg, Nike+, Sworkit) of their choice. Waitlist control participants had access to recommended mobile apps and an invitation to join an exercise group after the 8-week study period. Main outcomes assessed included changes in self-reported moderate, vigorous, and moderate to vigorous physical activity (MVPA) minutes per week in aggregate and stratified by whether women met Centers for Disease Control and Prevention guidelines for sufficient aerobic activity at baseline. Outcomes were measured through self-assessed Web-based questionnaires at baseline and 8 weeks.

**Results:**

The intervention was effective at increasing exercise for inactive women and proved to be feasible and acceptable to all participants. A total of 64 women were randomized, 30 to intervention and 34 to control. Women attended 2.8 sessions per week. There was a strong, but not statistically significant, trend toward increasing moderate, vigorous, and MVPA minutes for all women. As hypothesized, in the prespecified stratum of women who were inactive at baseline (n=51), intervention participants significantly increased their activity by an average of 50 (95% CI 4.0-95.9, P=.03) MVPA minutes per week more than control participants. They had a corresponding statistically significant net increase of 19 (95% CI 3.2-34.8, P=.02) minutes of vigorous activity. Inactive women in the intervention arm also experienced promising reductions in depression, reporting a statistically significant net decrease in their depression score (−3.8, 95% CI −7.0 to −0.6; P=.02).

**Conclusions:**

We found that a group exercise intervention using videoconferencing and mobile apps was a feasible and acceptable way to deliver a physical activity intervention to mothers. The intervention increased physical activity in inactive mothers. Further studies are needed to better establish how long these changes in physical activity can be maintained and whether these findings can be reproduced in a more diverse population.

**Trial Registration:**

ClinicalTrials.gov NCT02805140; https://clinicaltrials.gov/ct2/show/NCT02805140 (Archived by WebCite at http://www.webcitation.org/6yYZwRveg)

## Introduction

### Background

Despite strong evidence of the health benefits of physical activity and decades of efforts to increase activity levels, almost half of the United States adult population fails to meet Centers for Disease Control and Prevention (CDC) exercise guidelines of 150 minutes of moderate or 75 minutes of vigorous exercise per week, and 70% of the population fails to meet the biweekly muscle strengthening guidelines [1-6]. One group with unique challenges to being sufficiently active is women with young children. Women significantly reduce their activity levels in the transition to motherhood [7-9]. Mothers are less likely to be active than fathers, women of the same age who do not have children, and compared with their own activity levels before having children [7]. The proportion of hours per week that mothers with young children are physically active has decreased by 14 hours per week in the past 45 years, whereas sedentary activities such as watching television and driving have increased by 6 hours per week [10]. This decrease in physical activity is not only a concern for the health of mothers but also for their potential impact on their children. Active mothers have a positive influence on the activity levels of their children [11-13]. In addition, when mothers exercise, they report being able to better manage the demands of raising children [14-17]. Due to mothers’ unique needs and risks, it is important that we design appropriate interventions to help mothers be more physically active.

Mothers experience a wide range of barriers to exercising including isolation, a lack of leisure time, lack of social support, lack of child care, lack of spousal support, and the need to put family obligations ahead of themselves [16-19]. Reviews suggest that 2 elements of effective physical activity interventions that can help overcome such barriers include (1) adapting to individual needs and (2) incorporating community-based social support [20,21]. Individually adaptive interventions are able to tailor to individuals’ needs, preferences, and contexts. Social support interventions often draw upon support and accountability that individuals within a network can provide one another. These elements have each been tested successfully in physical activity interventions with mothers, but their combined impact is not known [7]. Unfortunately, individually adaptive and group physical activity interventions can be costly and complicated to deliver, and in-person groups can be particularly inconvenient for mothers.

Digital technology interventions represent a convenient, cost-effective, and scalable delivery mechanism for providing socially supportive and individually adaptive interventions [22]. In the United States, 77% of the adult population owns a mobile phone, and this proportion continues to increase rapidly [23]. More than half of downloaded apps are in the health and fitness domain, yet few exercise apps incorporate evidence-based content [24-28]. Mothers, in particular, are heavy users of technology, and thus represent an important group to test evidence-based technology interventions [29]. Technology interventions have a growing evidence base for being effective at increasing activity, though this research is in its early stages [30-33]. Additionally, videoconferencing tools such as Google Hangouts and Skype have been tested for exercise video coaching but not as a way to bring participants, and mothers specifically, together for real-time exercise video groups [34].

### Objectives

In this study, we assessed the feasibility and acceptability and estimated the effectiveness of a group physical activity intervention that incorporated videoconferencing and exercise mobile apps. This intervention relied on providing evidence-based elements of social support and individualization to increase physical activity in mothers.

## Methods

### Study Design

The Moms Online Video Exercise (MOVE) Study was an 8-week, 2-armed, parallel, Web-based randomized trial comparing the effectiveness of an intervention arm consisting of exercise groups that used videoconferencing and mobile apps with a waitlist control arm. We detailed our methods below and in an eHealth checklist (Multimedia Appendix 1).

### Recruitment

We recruited participants using advertisements that included a link to our study website in parent-specific Facebook groups and email listservs. Participants were recruited from all over the country, though the recruiting efforts and time zones available were targeted to the West Coast. In addition to email and Facebook advertisements, all recruited participants were asked to share the advertisement with any relevant email listservs or Facebook groups and any individuals they thought might be interested. Once on the study website, women were able to sign up for an introductory phone call in which study staff reviewed study procedures and consent forms using DocuSign (DocuSign, California, USA) before enrollment began. Recruitment efforts took place between July 2016 and November 2016. Before recruitment, we received approval from the University of California, San Francisco Institutional Review Board (14-15344), and registered our trial with the Clinical Trials Registry (NCT02805140).

### Participants

Our eligibility criteria stipulated that women needed to be between the ages of 18 and 60 years, speak and understand English, be able to give consent, and have at least 1 child under the age of 12 years. Enrolled women could not be pregnant or plan on being pregnant during the study period. Participants were also required to have access and understand how to operate 2 devices, one with videoconferencing capacity and one with mobile app capacity. These devices could include cell phones, computers, and smart tablets. Participants had to be capable of exercising safely, which was assessed using the validated Physical Activity Readiness Questionnaire [35]. We targeted women who were inactive (did not meet CDC physical activity guidelines), but hypothesized that even physically active women with young children might benefit from the intervention, and hence included them, but planned a priori to analyze them separately.

### Protocol

Women who were eligible for the study were asked to complete one introductory phone call, a baseline survey, and a practice group exercise video session to be randomized. Informed consent was obtained by study staff in the introductory phone call, and consent forms were electronically signed during or after the call. In practice group video sessions, participants signed into Google hangouts, introduced themselves, and then opened up a mobile app to complete a short workout using the Johnson & Johnson mobile app 7-minute workout routine [36]. Participants who confirmed their continued interest in participating in the study and who completed a baseline survey were randomized to the intervention or waitlist control. All participants were provided with access to a list of recommended mobile exercise apps. Women randomized to the intervention were additionally assigned to a video exercise group at a time of their preference and provided an exercise prescription. The exercise prescription over 8 weeks for intervention participants consisted of 5 weekday video exercise sessions lasting between 5 and 30 minutes and varying in type (interval training, dance, yoga, etc) and intensity (low to high) depending on the participant’s choice of mobile app and associated routine for each session. Adherence to this prescription was monitored via self-report, and staff support was provided if needed via email. After the 8-week study period, all participants were asked to fill out end of study surveys. Women in the intervention arm were then given the option of continuing for an additional 8 weeks, whereas those in the waitlist control were invited to join an exercise group for 8 weeks. The main analysis included only data from the 8-week study period, during which the waitlist control participants were not participating in video sessions.

Before randomization, women were asked to pick a morning time slot that they could attend every weekday for 8 weeks. We offered exercise group time slots on the half hour from 6:00 AM to 9:30 AM Pacific Standard Time Zone (PST). Exercise group sizes ranged from 2 to 5 participants. We enrolled participants over a period of 5 months. Groups grew over time as new participants enrolled and those from the waitlist arm joined groups after their 8-week waiting period. Group sessions lasted no more than a total of 30 minutes, beginning with a check-in lasting up to 5 minutes. Participants had an individualized website that contained a link to their respective Google Hangouts videoconferencing group calls and a tracking form that they filled out before each session (Multimedia Appendix 2). Women usually did their workouts while remaining on video to provide accountability (passive monitoring) and support (solidarity from working out simultaneously), much like having a “gym buddy.” We recommended freely available mobile apps and YouTube exercise videos routines that were updated on our study website as the study progressed (Multimedia Appendix 3). Participants were also encouraged to find exercise mobile apps and videos that were not on the list. Women were encouraged to individualize their choice of workout during a session, so participants in groups were often performing a wide range of workouts simultaneously. One of the study goals was to assess whether the hypothesized benefits in accountability and support would still be obtained while providing individual choice of workout routines. In addition to group exercise sessions, participants were connected to their group members via email primarily so they could communicate about planned or unplanned absences. Study staff monitored attendance via tracking forms that were filled out at each session and reached out to participants who had missed more than a week of workout sessions to check in over email.

### Randomization

Participants were randomized using parallel arms, equal allocation (1:1), and block randomization (random block sizes of 2 and 4 participants). The randomization was stratified on the participant’s morning time slot of choice and the participant’s baseline activity status, a binary variable of whether they met CDC guidelines of 150+ moderate to vigorous physical activity (MVPA) minutes per week. We stratified on these 2 factors to address potential confounding by baseline activity status and to ensure evenly sized intervention and control arms within time strata. Our statistician generated a stratified block random sequence using Stata 14 (StataCorp, Texas, USA) and stored it in Research Electronic Data Capture (REDCap), a secure, Web-based database application hosted at the University of California, San Francisco [37]. The sequence was concealed from the primary investigator who used REDCap to reveal the computer-assigned randomization once participants were enrolled. The assignment was not blinded to investigators or participants.

### Measures

#### Study Measurement Procedure

During the recruitment phase, participants filled out a screening survey to establish eligibility. Once eligibility was confirmed by study staff, participants were asked to complete a self-assessed baseline survey that included primary and secondary outcomes. At 8 weeks, all trial participants were asked to fill out self-assessed questionnaires with the same outcomes. Mothers who were randomized to the intervention arm were asked additional evaluation questions. All surveys were Web-based and completed online using Qualtrics (Qualtrics, Utah, USA) software.

#### Physical Activity

We assessed our primary outcome of physical activity using a self-assessed validated questionnaire, the Active Australia Survey [38,39]. Participants reported the frequency and duration of the past 7 days of activity in the following categories: walking (for at least half a mile), moderate activity (makes you breathe harder than normal), and vigorous activity (makes you sweat, out of breath). MVPA minutes per week were calculated by the sum of vigorous minutes multiplied by a factor of 2 plus the number of moderate minutes. The Active Australia Survey has good reliability and good validity compared with accelerometry and was found to be responsive to change in clinical trials [40,41]. Furthermore, it has been used in a number of physical activity trials with mothers [42,43].

#### Secondary Measures and Study Evaluation

We collected a self-report of weight. We assessed psychosocial measures specific to physical activity, which included social support for physical activity and physical activity self-efficacy [42,44,45]. We used Patient-Reported Outcome Measurement Information System (PROMIS) short form measures for anxiety, sleep disturbance, depression, and fatigue, and converted summary scores into standardized T-scores [46]. PROMIS T-scores are a standardized score based on a mean of 50 and a standard deviation of 10 using the reference population of a sample of the 2000 General Census [47-50]. We assessed participant adherence by monitoring their session attendance per week throughout their 8-week participation. Adherence took into account holiday weeks; the rate for the week excluding the holiday was applied to the whole holiday week. Acceptability was assessed through survey evaluation questions administered to participants in the intervention arm at the end of the study.

### Statistical Analysis

We used an intention-to-treat analysis. We analyzed all women who completed baseline and 8-week surveys (complete cases) according to their randomization status. On the basis of our a priori hypothesis that inactive women would benefit most from the study, we analyzed results for all women who completed 8-week surveys, followed by an analysis stratified by whether women met CDC aerobic guidelines (150+ minutes of MVPA per week) at baseline. We used linear regression to compare changes in minutes per week of physical activity from baseline to 8 weeks across randomized arms for the following categories: MVPA, moderate, and vigorous minutes per week. We included the following additional covariates in our model: baseline value of the outcome and the timeslot at which women chose to join their sessions. Time was included as an 8-part variable (time slots from 6:00 AM to 9:30 AM PST) and included in the model using dummy variables. We did not include time as a covariate in models for women who met activity guidelines due to inadequate sample size. We used these same linear regression models and covariates to analyze secondary outcomes of changes in weight and psychosocial measures. We assessed recruitment and retention rates, adherence (measured by attendance of video sessions in the intervention arm), and acceptability (through questionnaire feedback from intervention participants). We carried out 4 sensitivity analyses for inactive and all mothers for the physical activity outcome measures of MVPA, moderate, and vigorous minutes per week. They included omitting time from the model, adjusting for total number of children which was imbalanced at baseline, replacing missing values assuming no change from baseline, and finally a “worst case scenario” where we replaced missing values with the respective randomization arm mean plus a standard deviation for control participants and minus a standard deviation for intervention participants. Our sample size was estimated based on informal pilot data where we found an average increase of 30 minutes per week (standard deviation of 15 minutes per week) in 5 adherent participants over 8 weeks using a single intervention arm. We calculated that we needed at least 32 participants to have 80% power (with alpha=0.05) to detect a 20-minute difference in MVPA minutes per week between randomization arms if attrition was less than 10%, and we assumed an increase of 10 minutes per week in the control arm. As we found that it was feasible to recruit more participants during the planned recruitment period, we exceeded the minimum number of participants we aimed to enroll based on these sample size calculations.

## Results

### Participant Characteristics

**Figure 1:**
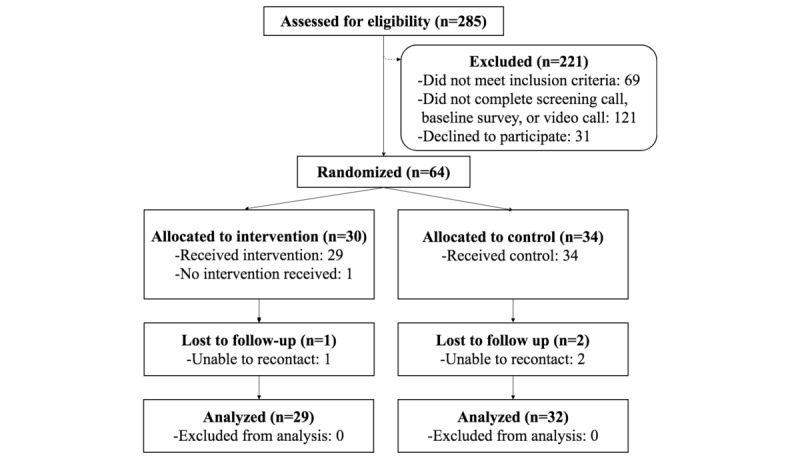
Participant flow diagram. One participant declined to participate after randomization to the intervention arm, and that same participant did not complete end-of-study assessments. For our main analysis, we included complete cases (n=61); we also completed 2 sensitivity analyses to assess the possible effects of missing data that included all randomized participants (n=64).

We randomized 64 participants who were recruited over 5 months (July 2016-November 2016), 30 were allocated to the intervention and 34 to the control arm (Figure 1). All participants completed baseline surveys in which the majority reported not meeting CDC activity guidelines (54/64, 84%). At the 8-week follow-up time (October 2016-January 2017), 3 out of 64 participants were unable to be contacted, 2 from the control arm and 1 from the intervention arm, resulting in an overall loss of 5% for all participants (in analyses stratified by baseline activity, this equated to a 6% (3/54) loss for the inactive group, to which all 3 missing participants belonged). Participants with complete data (n=61) were included in analyses of primary, secondary, adherence, and feasibility outcomes.

The mean age of all women enrolled in the trial (n=64) was 37 years, and on average, they had less than 2 children (Table 1). Participants were predominately married, white, and had a high level of education, the majority with a post-graduate degree. Most women worked full-time or part-time jobs.

### Physical Activity

Mothers in the intervention arm (n=29) increased their mean number of MVPA minutes per week by 42.2 (95% CI −11.3 to 95.7, *P*=.12) more minutes than mothers in the control arm (n=32), adjusted for baseline MVPA minutes per week and group time slot (Table 2). The intervention arm mothers increased moderate activity by 13.8 (95% CI −4.8 to 32.4, *P*=.14) more minutes per week and vigorous activity by 13.2 (95% CI −7.3 to 33.8, *P=*.20) more minutes per week.

**Figure 2:**
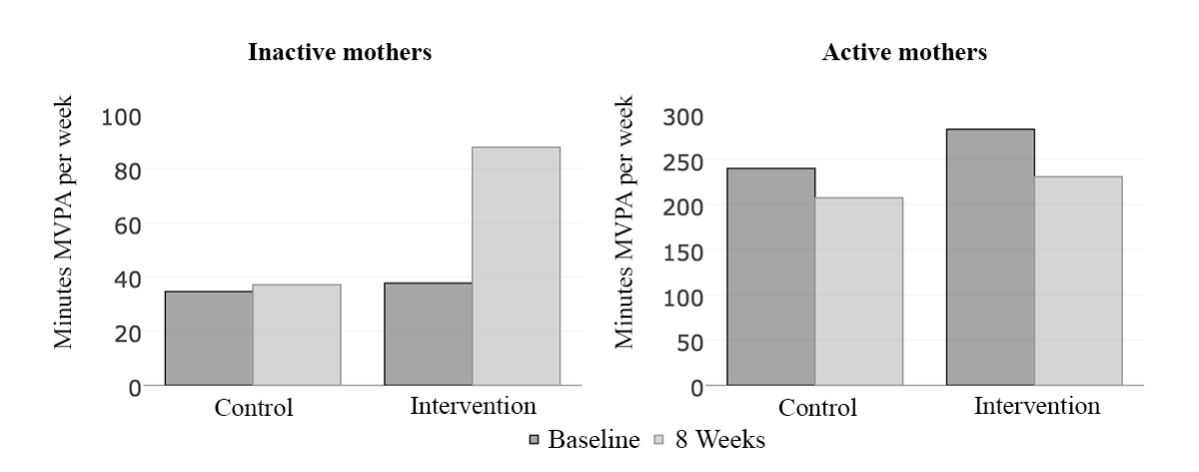
Moderate to vigorous physical activity (MVPA) minutes per week at baseline and 8 weeks for inactive (n=51) and active (n=10) women. MVPA minutes are calculated as follows: moderate minutes+(2×vigorous minutes). Unadjusted minutes of MVPA per week at baseline are in dark gray and at 8 weeks in light gray.

Mothers who were inactive at baseline (n=51) increased their MVPA minutes per week by 50 (95% CI 4.0- 95.9, *P=*.03) more minutes in the intervention arm (n=23) compared with the control arm (n=28; see Table 2 and Figure 2). Inactive mothers at baseline assigned to the intervention arm increased their vigorous minutes per week by a net 19 (95% CI 3.2- 34.8, *P=*.02) minutes compared with the controls and increased their moderate activity minutes by 9.7 (95% CI −11.3 to 30.7, *P=*.36) min. In contrast, we found no statistically significant differences in physical activity outcomes across arms in mothers who were active at baseline (n=10).

### Post Hoc Sensitivity Analysis

Our sensitivity analyses indicated that our physical activity models for all and inactive mothers were neither sensitive to omitting exercise time slot as a covariate in the model nor to various strategies for imputing the values of missing data (n=3, see Methods for a description of the imputation approaches). However, the estimated effect of treatment on MVPA in inactive women was attenuated and no longer statistically significant after adjustment for how many children women had, a baseline variable which was imbalanced across randomization arms. Women had more children in the control arm, and having additional children was independently associated with lower activity levels. However, the significant treatment effect on vigorous activity in inactive women was not sensitive to adjustment for this imbalance in number of children across arms (Multimedia Appendix 4).

### Secondary Outcomes

We examined changes in several secondary outcomes: weight, social support for physical activity, physical activity self-efficacy, and 4 health-related quality of life measures (Table 3). Among women who were inactive at baseline, the intervention arm lost 2 (95% CI −4.2 to 0.2) more kilograms than the control arm (*P=*.07). Social support for physical activity increased more for women in the intervention arm than in the control arm (*P=*.04). The difference in physical activity self-efficacy changes across trial arms was not statistically significant (*P=*.80).

Women in the intervention arm compared with the control arm did not experience statistically significant changes in the health-related quality of life measures of sleep, anxiety, fatigue, or depression across the 8 weeks. In stratified analyses, inactive women had a greater decrease in their depression score, a statistically significant difference of −3.8 (95% CI −7.0 to −0.6, *P=*.02).

### Adherence and Acceptability

Women in the intervention arm (n=30) attended 2.8 group video sessions per week on average for over 8 weeks. The attendance had a standard deviation of 1.17 and a skewed distribution with a median of 3.5. Participants attended 3.3 sessions per week in the first half of the study and 2.4 sessions per week in the second half. Moreover, 5 participants from the intervention arm did not complete the entire 8 weeks, though all, except 1, completed end-of-study assessments. Women reported multiple reasons for noncompletion, including work, ill health, and lack of sleep.

**Table 1:** Baseline characteristics.

Characteristic		Control (n=34)^a^	Intervention (n=30)^a^
Mother’s age in years, mean (SD)		36.8 (6.5)	37.3 (4.0)
Children’s age in years, mean (SD)		2.5 (1.9)	2.9 (2.1)
Number of children, mean (SD)		1.8 (0.8)	1.4 (0.5)
**Marital status^b^, n (%)**			
	Married or living as married	29 (88)	28 (93)
	Never married	3 (9)	1 (3.3)
	Separated or divorced	1 (3)	1 (3)
**Race ethnicity, n (%)**			
	African American	1 (3)	1 (3)
	Asian	4 (12)	3 (10)
	Latina	0 (0)	1 (3)
	Middle Eastern	2 (6)	0 (0)
	Two or more races^c^	5 (15)	4 (13)
	White	22 (65)	21 (70)
**Employment, n (%)**			
	Full time	17 (50)	18 (60)
	Not employed	9 (27)	4 (13)
	Part time	7 (21)	6 (20)
	Student	1 (3)	2 (7)
**Education level, n (%)**			
	Some college	1 (3)	1 (3)
	Bachelor degree	9 (27)	10 (33)
	Post college degree	24 (71)	19 (63)
Currently breastfeeding, n (%)		15 (44)	12 (40)
**Physical activity in minutes per week, mean (SD)**			
	Moderate to vigorous	59.1 (80.1)	89.5 (112.5)
	Vigorous	13.5 (29.6)	24 (44.8)
	Moderate	32.1 (38.2)	41.5 (50.3)
BMI^d^ (kg/cm^2^), mean SD)		24.1 (3.3)	25.6 (4.6)
**Physical activity measures in score, mean (SD)**			
	Physical activity self-efficacy	3.5 (0.7)	3.6 (0.7)
	Physical activity social support	2.0 (0.6)	2.1 (0.8)
**PROMIS^e^ measures in T-score^f^, mean (SD)**			
	Depression	48.2 (6.8)	48.7 (7.6)
	Sleep	57.7 (7.6)	57 (6.9)
	Fatigue	60.0 (8.7)	59.4 (6.0)
	Anxiety	50.4 (9.2)	51.9 (7.9)

^a^We used stratified randomization (time and baseline activity status), which resulted in intervention and control arms of unequal sizes.

^b^One person (from control arm) chose not to answer.

^c^Race/ethnicity—Two or more races category includes the following (n): Latina/white (1), Latina/Middle Eastern (1), Middle Eastern/white (3), Asian/white (2), American Indian/white (1), Mixed race-not specified (1).

^d^BMI: body mass index.

^e^PROMIS: Patient-Reported Outcome Measurement Information System.

^f^See Methods for scoring of PROMIS measures

**Table 2:** Changes in minutes of physical activity over 8 weeks by randomization arm and differences in changes across randomization arms.

Outcome measures		8-week change in physical activity minutes per week^a^ (95% CI)		Difference across arms in 8-week changes^a,b^ (95% CI)	*P* value
		Control arm	Intervention arm		
**All mothers (n=61)**					
	MVPA^c^	−7.3 (−43.7 to 29.2)	34.9 (−3.4 to 73.2)	42.2 (−11.3 to 95.7)	.12
	Vigorous	0.9 (−13.0 to 14.9)	14.2 (−0.5 to 28.8)	13.2 (−7.3 to 33.8)	.20
	Moderate	−8.2 (−21.0 to 4.5)	5.6 (−7.8 to 19)	13.8 (−4.8 to 32.4)	.14
**Inactive mothers (n=51)**					
	MVPA^c^	1.5 (−29.3 to 32.2)	51.4 (17.5-85.4)	50.0 (4.0-95.9)	.03
	Vigorous	−0.1 (−10.7 to 10.4)	18.9 (7.2-30.5)	19.0 (3.2-34.8)	.02
	Moderate	2.8 (−11.2 to 16.8)	12.5 (−3 to 27.9)	9.7 (−11.3 to 30.7)	.36
**Active mothers (n=10)**					
	MVPA^c^	−9.1 (−204 to 185.8)	−68.1 (−225.5 to 89.3)	−59.0 (−315.9 to 197.9)	.60
	Vigorous	10.8 (−81.8 to 103.3)	−5.5 (−81.0 to 70.0)	−16.3 (−135.9 to 103.3)	.76
	Moderate	−65.3 (−110.6 to −20.0)	−34.0 (−70.5 to 2.5)	31.3 (−28.7 to 91.3)	.26

^a^Adjusted for baseline value of outcome and exercise time slot of choice for all mothers and inactive mothers. Adjusted for baseline value of outcome for active mothers.

^b^Difference of the within-group change for intervention versus control arm.

^c^MVPA: moderate to vigorous physical activity.

**Table 3:** Differences across randomization arms in changes in secondary outcomes over 8 weeks.

Outcome measures		All mothers (n=61)		Inactive mothers (n=51)		Active mothers (n=10)	
		Treatment effect^a^ (95% CI)	*P* value	Treatment effect^a^ (95% CI)	*P* value	Treatment effect^b^ (95% CI)	*P* value
Weight loss (kg)		−1.6 (−3.7 to 0.6)	.15	−2.0 (−4.2 to 0.2)	.07	−2.6 (−18.2 to 12.9)	.69
Physical activity social support^c^		0.3 (0.0-0.6)	.04	0.2 (−0.1 to 0.5)	.15	0.9 (0.1-1.7)	.04
Physical activity self efficacy^c^		0.0 (−0.3 to 0.2)	.80	−0.1 (−0.3 to 0.2)	.71	0.0 (−0.6 to 0.6)	.87
**PROMIS^d^ measures**							
	Depression^e^	−3.0 (−5.9 to 0.0)	.05	−3.8 (−7.0 to −0.6)	.02	2.5 (−7.2 to 12.3)	.56
	Sleep disturbance^e^	−1.3 (−3.8 to 1.2)	.32	−1.2 (−4.0 to 1.6)	.40	−0.3 (−8.1 to 7.6)	.94
	Fatigue^e^	−1.1 (−5.1 to 2.8)	.57	−1.6 (−5.6 to 2.5)	.44	0.5 (−13.5 to 14.5)	.93
	Anxiety^e^	−1.1 (−4.5 to 2.2)	.50	−1.3 (−5.2 to 2.7)	.52	1.6 (−6.5 to 9.7)	.66

^a^Adjusted for baseline value of outcome and exercise time slot of choice.

^b^Adjusted for baseline value of outcome for active mothers.

^c^Higher scores indicate a more optimal outcome.

^d^PROMIS: Patient-Reported Outcome Measurement Information System.

^e^Lower scores indicate a more optimal outcome.

**Satisfaction with the Moms Online Video Exercise (MOVE) intervention—participants’ qualitative survey assessments.**

**Things we liked best**

- “That it got me doing SOMETHING physical which I really, really needed.”
- “I liked having the time set out for me to do the workout and having other people ‘keeping me company.’ That was a HUGE motivator.”
- “Creating a structured time for myself and following through.”
- “Loved the group motivation.”
- “Working out from home, having accountability, the “come as you are” mentality, the other gals were great!”
- “I discovered that 15 minutes of morning exercise made my body feel better immediately and often for the rest of the day.”
- “The workouts. You really can notice results with 15 minutes per day.”
- “The ‘live’ nature of the sessions.”
- “Knowing that there were other moms in the same boat as me.”
- “Having a program to participate in created more support from [my] partner around exercise.”

**Things we would change**

- “Some way to help push yourself to increasingly challenging programs in a measured way.”
- “More workout options.”
- “Offer more flexibility in the time.”
- “Better introductions when a new person starts.”
- “It would be nice to be able to join a later group if we can’t make our regularly scheduled group.”

The majority of mothers (86%) expressed satisfaction (extremely or somewhat satisfied) with the intervention. All mothers said they would recommend it to a friend, either certainly (96%) or maybe (4%). Mothers reported that the most significant impact from their participation was increasing their fitness levels (36%), being a good role model for their kids (14%), improving mood (11%), and feeling better about their body (7%). The most frequently (sometimes and often) used apps and YouTube videos included Sworkit, Yoga YouTube videos, Johnson and Johnson, and Nike+. All women reported feeling a benefit after sessions, for example, “energized,” “great!,” and “proud.” A little less than half of the women in the intervention arm (42.9%) reported increasing their activity levels outside of the study and described these increases as: “The kids wanted to start doing more yoga (Cosmic Kids on YouTube) and dance parties as a family” and “I had more energy to do other activities throughout the day.” Most women reported that their biggest barriers to attendance were lack of sleep, family commitments, and work commitments. Most women (78%) reported in the survey that their commitment to the group and the expectation that others would be there and rely on them being present were the main motivators to attending sessions. In open survey responses to why participants liked the study, most listed social support, accountability, and convenience as their favorite features, as well as ones they would like further strengthened in future iterations of the program (Textbox 1).

## Discussion

### Summary of Results

The MOVE trial assessed an exercise group intervention using videoconferencing and mobile apps for mothers over an 8-week period through a randomized controlled design. The intervention was feasible and acceptable to participants. There was a trend toward increasing MVPA, moderate, and vigorous minutes of physical activity per week for all women, although this did not reach statistical significance. As hypothesized, women in the prespecified stratum who were inactive at baseline statistically significantly increased their MVPA minutes by an average of 50 minutes per week more in the intervention arm. A corresponding statistically significant increase of 19 minutes of vigorous activity drove the increase in total MVPA minutes per week for inactive women.

### Feasibility and Acceptability

Digital tools were the driving force behind the feasibility and acceptability of this intervention. Recruitment, enrollment, data collection, and intervention delivery were all conducted remotely using technology, which was convenient for participants and study staff. Programs that can adapt to the individual context of their participants and ones that provide strong social support have proved effective at increasing physical activity [20,21]. The digital tools we used helped us address individual needs of participants while creating a socially supportive exercise space. Mobile apps allowed participants to choose short, and often, vigorous workouts, which could be customized to individual abilities and interests. Using mobile exercise apps provided participants with a way of efficiently exercising without having to make major changes to their existing routines. Women exercised from the convenience of their home at the time of their choosing, usually alongside their children. The videoconferencing tools helped create a supportive social group, which enabled women to check in face-to-face regularly, and facilitated accountability. Simultaneous exercise sessions also provided a sense of solidarity, even when the individual exercise routines were not coordinated within groups.

The participants’ enthusiasm for the program was important in the early recruitment efforts, where participants shared study advertisements with multiple types of mother support group networks, and in the retention of participants who almost uniformly filled out end of study surveys, even if they no longer were able to participate in sessions. Many physical activity trials for mothers require fairly high time commitments from participants, primarily through coaching and education in person [43,51,52], remotely via telephone and texts [53,54] or both [55]. Participants’ time in this study went almost entirely toward exercising. Participants reported a strong appreciation for the convenience and flexibility of the intervention, which are particularly important features for mothers of young children who report feeling overwhelmed and unable to prioritize their own self-care [15,17-19,56]. Accordingly, our retention rates of 94% to 95% were higher than rates for 2 comparable technology trials on physical activity with mothers at equivalent time points of 86% at 1 month [53], 87% at 13 weeks [55], and among the highest of physical activity trials with mothers [43,51,52,54]. The high feasibility and acceptability of this trial has implications for future Internet physical activity trials targeting mothers.

### Effectiveness

Randomized trials of physical activity with mothers have mixed results. Some trials have found statistically significant increases in physical activity [43,51-53,55], whereas others report nonstatistically significant changes [54]. There is great heterogeneity in the types of interventions delivered and even inconsistency in the definition of MVPA. Some studies use a simple equation [moderate + vigorous = MVPA], whereas others use a vigorous enhanced equation [moderate + vigorous × 2 = MVPA] as used in these analyses. Moreover, 2 comparable randomized technology trials of physical activity with mothers that incorporated technology found statistically significant increases in MVPA minutes in the range of the increases we found in inactive women [53,55]. One trial that utilized a physical activity website with resources, pedometers, and telephone counselors to provide motivational interviewing to help mothers incrementally work up to a goal of 150 MVPA found an increase of 92 MVPA minutes per week for mothers of babies 3+ months comparable with our difference of 50 MVPA minutes per week using the vigorous enhanced equation; a second trial that had mothers set their own exercise goals and provided support in meeting these goals over 13 weeks via individually tailored text messages found an increase of 49 MVPA minutes per week comparable with our difference of 30 MVPA minutes per week using the simple equation. These 2 studies were larger and longer and they differed from this study in that they had a large coaching component, did not include any group social support, and did not use apps or videoconferencing tools. Changes in vigorous minutes were not disaggregated from MVPA minutes per week in either of these studies.

### Secondary Findings

In addition to the increases in physical activity, we observed improvements in several secondary measures. Social support specific to physical activity increased for mothers in this trial. Mothers have a uniquely challenging set of barriers to physical activity. Our participants reported that they were motivated to show up for one another (social support), and the presence of other mothers re-enforced their own capacity to exercise consistently (self-efficacy). We observed a statistically significant decrease in depression among inactive women in the intervention arm across the trial period. The increases in physical activity and social support that we observed could both contribute to decreased depression [4,57]. These are mechanisms that could be tested individually and synergistically in future trials.

### Limitations

Our digital tools helped create an efficient recruiting process; however, our recruitment and enrollment strategies and inclusion criteria resulted in a sample that was not representative of the United States population. Our requirements of completing a phone call, survey, and a practice video session before randomization could have produced a sample that was more adherent than the general population. Furthermore, recruitment strategies that targeted mothers’ groups through Facebook and email as well as the inclusion criteria that required an ability to own and use 2 devices while potentially caring for a child could have contributed to our sample consisting primarily of women who were highly educated, married, white, of an older age at first child, and typically lived in large cities on the West Coast. Future trials are needed to test whether this type of intervention could be replicated in a more diverse population.

We relied on a self-report measure of physical activity, which though validated and widely used, could have introduced bias. Participants and investigators were not blinded to their randomization status, which could have also introduced bias. Our sample size limited our ability to fully explore the differences in outcomes by baseline activity status. In particular, the group of mothers who were physically active at baseline was quite small (n=10). Although our results clearly suggest that this type of intervention is most likely to benefit mothers who are inactive, it would be premature to conclude, based on our data, that this approach does not benefit all mothers.

In post hoc sensitivity analyses, we found that our model was neither sensitive to the removal of exercise time slot nor to 2 imputation strategies to address missing data. However, we did find that our results for MVPA among inactive women were sensitive to adjustment for number of children, which was imbalanced at baseline. In contrast, results for vigorous activity retained statistical significance, suggesting that our intervention may be most effective at increasing higher intensity activity. Although the imbalance resulted by chance in this small sample, stratification of the randomization by number of children could be worth exploring in future studies in this population.

We were unable to test a longer intervention due to limited resources, and we could not assess whether the intervention effect could be maintained over a longer time due to our waitlist design. In addition, we were unable to fully disentangle the impact of videoconferencing separate from the impact of mobile apps. The waitlist group participated in a required initial videoconferencing session using a mobile app and was given access to the recommended list of apps, which suggests that the videoconferencing drove the difference between randomization arms; however, the intervention arm additionally had a specific prescription of exercise as well as support and monitoring by study staff to meet the prescription, which limits our ability to attribute any differences solely to videoconferencing.

### Conclusions

This study suggests that technology can be used to create an individualized physical activity intervention with social support using a scalable and cost-effective delivery mechanism for mothers. There is great excitement in the use of new technology to solve old problems; however, often new technology alone cannot overcome the barriers to behavior change. We utilized technology to deliver evidence-based components of individualization and social support in a physical activity program that was convenient and compelling for our busy participants. To our knowledge, this is the first study that examines the use of videoconferencing paired with exercise mobile apps to create exercise groups. We found that using videoconferencing and mobile apps was a feasible and acceptable way to deliver a physical activity group intervention for mothers. Furthermore, we showed our intervention increased physical activity in inactive mothers. Further studies are needed to better establish how long these changes in physical activity can be maintained and whether these findings can be reproduced in a more diverse population.

## Appendix

Multimedia Appendix 1 CONSORT‐EHEALTH checklist (V 1.6.1).

Multimedia Appendix 2 Participant personal website.

Multimedia Appendix 3 Recommended exercise apps and websites.

Multimedia Appendix 4 Post hoc sensitivity analyses—differences across randomization arms in physical activity changes over 8 weeks for inactive mothers and all mothers.

## Abbreviations

CDC: Centers for Disease Control and Prevention
MOVE: Moms Online Video Exercise
MVPA: moderate to vigorous physical activity
