# Supplementary material for: Increasing Physical Activity in Mothers Using Video Exercise Groups and Exercise Mobile Apps: Randomized Controlled Trial
Source: J Med Internet Res. 2018 May 18;20(5):e179. doi: 10.2196/jmir.9310 (PMC5984277; doi:10.2196/jmir.9310)
Supplement: Multimedia Appendix 1 [file jmir_v20i5e179_app1.pdf]

CONSORT-EHEALTH Checklist V1.6.2 Report	Manuscript Number	9310
**(based on CONSORT-EHEALTH V1.6), available at [http://tinyurl.com/consort-ehealth-v1-6].**		
**Date completed**		
5/15/2018 14:29:55		
**by**		
Maya Mascarenhas		
Increasing Physical Activity in Mothers Using Video Exercise Groups and Exercise Mobile Apps: A Randomized Controlled Trial		
TITLE		
**1a-i) Identify the mode of delivery in the title**		
"...Using Video Exercise Groups and Exercise Mobile Apps..."		
**1a-ii) Non-web-based components or important co-interventions in title**		
NA all important cointerventions listed above in mode of delivery		
**1a-iii) Primary condition or target group in the title**		
"Increasing Physical Activity in Mothers..."		
ABSTRACT		
**1b-i) Key features/functionalities/components of the intervention and comparator in the METHODS section of the ABSTRACT**		
The Moms Online Video Exercise Study was an 8-week, 2-armed, Web-based randomized trial comparing the effectiveness of a group exercise intervention with a waitlist control.		
**1b-ii) Level of human involvement in the METHODS section of the ABSTRACT**		
Apart from onboarding and occasional email checkins from study staff with participants via email, the intervention was entirely participant driven.		
**1b-iii) Open vs. closed, web-based (self-assessment) vs. face-to-face assessments in the METHODS section of the ABSTRACT**		
"Outcomes were measured through self-assessed Web-based questionnaires at baseline and 8 weeks."		
**1b-iv) RESULTS section in abstract must contain use data**		
"A total of 64 women were randomized, 30 to intervention and 34 to control. Women attended 2.8 sessions per week. There was a strong, but not statistically significant, trend toward increasing moderate, vigorous, and MVPA minutes for all women. As hypothesized, in the prespecified stratum of women who were inactive at baseline (n=51), intervention participants significantly increased their activity by an average of 50 (95% CI 4.0- 95.9, P=.03) MVPA minutes per week more than control participants. They had a corresponding statistically signicant net increase of 19 (95% CI 3.2-34.8, P=.02) minutes of vigorous activity. Inactive women in the intervention arm also experienced promising reductions in depression, reporting a statistically significantcant net decrease in their depression score (−3.8, 95% CI −7.0 to −0.6; P=.02)."		
**1b-v) CONCLUSIONS/DISCUSSION in abstract for negative trials**		
"We found that a group exercise intervention using videoconferencing and mobile apps was a feasible and acceptable way to deliver a physical activity intervention to mothers. The intervention increased physical activity in inactive mothers."		
INTRODUCTION		
**2a-i) Problem and the type of system/solution**		
"Due to mothers' unique needs and risks, it is important that we design appropriate interventions to help mothers be more physically active."		
**2a-ii) Scientific background, rationale: What is known about the (type of) system**		
"Mothers, in particular, are heavy users of technology, and thus represent an important group to test evidence-based technology interventions [29]. Technology interventions have a growing evidence base for being effective at increasing activity, though this research is in its early stages [30-33]. Additionally, videoconferencing tools such as Google Hangouts and Skype have been tested for exercise video coaching but not as a way to bring participants, and mothers speficically, together for real-time exercise video groups [34]"		
**Does your paper address CONSORT subitem 2b?**		
"In this study, we assessed the feasibility and acceptability and estimated the effectiveness of a group physical activity intervention that incorporated videoconferencing and exercise mobile apps. This intervention relied on providing evidence-based elements of social support and individualization to increase physical activity in mothers."		
METHODS		

**3a) CONSORT: Description of trial design (such as parallel, factorial) including allocation ratio**		
"The Moms Online Video Exercise (MOVE) Study was an 8-week, 2-armed, parallel, Web-based randomized trial comparing the effectiveness of an intervention arm consisting of exercise groups that used videoconferencing and mobile apps with a waitlist control arm."		
**3b) CONSORT: Important changes to methods after trial commencement (such as eligibility criteria), with reasons**		
NA		
**3b-i) Bug fixes, Downtimes, Content Changes**		
No bugs or downtimes. The only content that changed was updated suggestions for exercise mobile apps as the study progressed.		
**4a) CONSORT: Eligibility criteria for participants**		
"Our eligibility criteria stipulated that women needed to be between the ages of 18 and 60 years, speak and understand English, be able to give consent, and have at least 1 child under the age of 12 years. Enrolled women could not be pregnant or plan on being pregnant during the study period. Participants were also required to have access and understand how to operate 2 devices, one with videoconferencing capacity and one with mobile app capacity."		
**4a-i) Computer / Internet literacy**		
"Participants were also required to have access and understand how to operate 2 devices, one with videoconferencing capacity and one with mobile app capacity. These devices could include cell phones, computers, and smart tablets."		
**4a-ii) Open vs. closed, web-based vs. face-to-face assessments:**		
Participants were recruited online via emails and directed to our website to sign up for the study. This was a purely web-based trial. Study team did get to know participants through introductory phone calls, an initial practice session prior to randomization, and through email follow ups based on adherence in the intervention arm.		
**4a-iii) Information giving during recruitment**		
"Once on the study website, women were able to sign up for an introductory phone call in which study staff reviewed study procedures and consent forms using DocuSign (DocuSign, California, USA) before enrollment began."		
**4b) CONSORT: Settings and locations where the data were collected**		
"All surveys were completed online using Qualtrics software."		
**4b-i) Report if outcomes were (self-)assessed through online questionnaires**		
Participants self-assessed outcomes using online surveys.		
**4b-ii) Report how institutional affiliations are displayed**		
The institutional affiliation (UCSF) was displayed at the top of all survey forms as well as on the homepage of the website to comply with UCSF IRB recommendations.		
**5) CONSORT: Describe the interventions for each group with sufficient details to allow replication, including how and when they were actually administered**		
**5-i) Mention names, credential, affiliations of the developers, sponsors, and owners**		
See Multimedia Appendix 3		
**5-ii) Describe the history/development process**		
NA		
**5-iii) Revisions and updating**		
NA		
**5-iv) Quality assurance methods**		
NA		
**5-v) Ensure replicability by publishing the source code, and/or providing screenshots/screen-capture video, and/or providing flowcharts of the algorithms used**		
The website is still live and provided here on this form at the top which will be a multimedia appendix to this paper.		
**5-vi) Digital preservation**		
We provided a screenshot of website in Multimedia Appendix.<br>"Participants had an individualized website that contained a link to their respective Google Hangouts videoconferencing group calls and a tracking form that they lled out before each session (Multimedia Appendix 1)." Web cached website is as follows: http://www.webcitation.org/6zQOQgXUu		
**5-vii) Access**		
"Participants had an individualized website that contained a link to their respective Google Hangouts videoconferencing group calls and a tracking form that they lled out before each session (Multimedia Appendix 2)."		
**5-viii) Mode of delivery, features/functionalities/components of the intervention and comparator, and the theoretical framework**		

" All participants were provided with access to a list of recommended mobile exercise apps. Women randomized to the intervention were additionally assigned to a video exercise group at a time of their preference and provided an exercise prescription."		
**5-ix) Describe use parameters**		
"The exercise prescription over 8 weeks for intervention participants consisted of 5 weekday video exercise sessions lasting between 5 and 30 minutes and varying in type (interval training, dance, yoga, etc) and intensity (low to high) depending on the participant's choice of mobile app and associated routine for each session."		
**5-x) Clarify the level of human involvement**		
"Adherence to this prescription was monitored via self-report, and staff support was provided if needed via email."		
**5-xi) Report any prompts/reminders used**		
"Adherence to this prescription was monitored via self-report, and staff support was provided if needed via email."		
**5-xii) Describe any co-interventions (incl. training/support)**		
All participants had to successfully complete a practice group video session, however, there was no training and minimal support for troubleshooting was provided.		
**6a) CONSORT: Completely defined pre-specified primary and secondary outcome measures, including how and when they were assessed**		
"We assessed our primary outcome of physical activity using a self-assessed validated questionnaire, the Active Australia Survey [38,39].<br>We collected a self-report of weight. We used Patient-Reported Outcome Measurement Information System (PROMIS) short form measures for anxiety, sleep disturbance, depression, and fatigue, and converted summary scores into standardized T-scores [46]."		
**6a-i) Online questionnaires: describe if they were validated for online use and apply CHERRIES items to describe how the questionnaires were designed/deployed**		
The Active Australia is validated and has been validated for self administration, though not for online. PROMIS measures have been validated for online use. Self efficacy and social support for physical activity have not been validated for online use.		
**6a-ii) Describe whether and how "use" (including intensity of use/dosage) was defined/measured/monitored**		
"We assessed participant adherence by monitoring their session attendance per week throughout their 8week participation. Adherence took into account holiday weeks, the rate for the week excluding the holiday was applied to the whole holiday week."		
**6a-iii) Describe whether, how, and when qualitative feedback from participants was obtained**		
"Acceptability was assessed through survey evaluation questions administered to participants in the intervention arm at the end of the study."		
**6b) CONSORT: Any changes to trial outcomes after the trial commenced, with reasons**		
"All surveys were completed online using Qualtrics software."		
**7a) CONSORT: How sample size was determined**		
**7a-i) Describe whether and how expected attrition was taken into account when calculating the sample size**		
"Our sample size was estimated based on informal pilot data where we found an average increase of 30 minutes per week (standard deviation of 15 minutes per week) in 5 adherent participants over 8 weeks using a single intervention arm. We calculated that we needed at least 32 participants to have 80% power (with alpha=0.05) to detect a 20-minute difference in MVPA minutes per week between randomization arms if attrition was less than 10%, and we assumed an increase of 10 minutes per gweek in the control arm."		
**7b) CONSORT: When applicable, explanation of any interim analyses and stopping guidelines**		
"We assessed our primary outcome of physical activity using a self-assessed validated questionnaire, the Active Australia Survey [38,39].<br>We collected a self-report of weight. We used Patient-Reported Outcome Measurement Information System (PROMIS) short form measures for anxiety, sleep disturbance, depression, and fatigue, and converted summary scores into standardized T-scores [46]."		
**8a) CONSORT: Method used to generate the random allocation sequence**		
"Our statistician generated a stratied block random sequence using Stata 14 (StataCorp, Texas, USA) and stored it in Research Electronic Data Capture (REDCap), a secure, Web-based database application hosted at the University of California, San Francisco [37]."		
**8b) CONSORT: Type of randomisation; details of any restriction (such as blocking and block size)**		
""Participants were randomized using parallel arms, equal allocation (1:1), and block randomization (random block sizes of 2 and 4 participants)."		
**9) CONSORT: Mechanism used to implement the random allocation sequence (such as sequentially numbered containers), describing any steps taken to conceal the sequence until interventions were assigned**		
"Our statistician generated a stratified block random sequence using Stata 14 (StataCorp, Texas, USA) and stored it in Research Electronic Data Capture (REDCap), a secure, Web-based database application hosted at the University of California, San Francisco [37]. The sequence was concealed from the primary investigator who used REDCap to reveal the computer-assigned randomization once participants were enrolled. The assignment was not blinded to investigators or participants."		

**10) CONSORT: Who generated the random allocation sequence, who enrolled participants, and who assigned participants to interventions**		
Statistician generated random sequence. Investigators enrolled patients and used RECAP to reveal randomization assignment.		
**11a) CONSORT: Blinding - If done, who was blinded after assignment to interventions (for example, participants, care providers, those assessing outcomes) and how**		
**11a-i) Specify who was blinded, and who wasn't**		
""The sequence was concealed from the primary investigator who used REDCap to reveal the computer-assigned randomization once participants were enrolled." The assignment was not blinded to investigators or participants."		
**11a-ii) Discuss e.g., whether participants knew which intervention was the "intervention of interest" and which one was the "comparator"**		
"The assignment was not blinded to investigators or participants."		
**11b) CONSORT: If relevant, description of the similarity of interventions**		
NA		
**12a) CONSORT: Statistical methods used to compare groups for primary and secondary outcomes**		
""We used an intention-to-treat analysis. We analyzed all women who completed baseline and 8-week surveys (complete cases) according to their randomization status. We used linear regression to compare changes in minutes per week of physical activity from baseline to 8 weeks across randomized arms for the following categories: MVPA, moderate, and vigorous minutes per week."		
**12a-i) Imputation techniques to deal with attrition / missing values**		
"We carried out 4 sensitivity analyses for inactive and all mothers for the physical activity outcome measures of MVPA, moderate, and vigorous minutes per week. They included omitting time from the model, adjusting for total number of children which was imbalanced at baseline, replacing missing values assuming no change from baseline, and nally a "worst case scenario" where we replaced missing values with the respective randomization arm mean plus a standard deviation for control participants and minus a standard deviation for intervention participants."		
**12b) CONSORT: Methods for additional analyses, such as subgroup analyses and adjusted analyses**		
"On the basis of our a priori hypothesis that inactive women would benet most from the study, we analyzed results for all women who completed 8-week surveys, followed by an analysis stratied by whether women met CDC aerobic guidelines (150+ minutes of MVPA per week) at baseline.<br>We included the following additional covariates in our model: baseline value of the outcome and the timeslot at which women chose to join their sessions."		
RESULTS		
**13a) CONSORT: For each group, the numbers of participants who were randomly assigned, received intended treatment, and were analysed for the primary outcome**		
"We randomized 64 participants who were recruited over 5 months (July 2016- November 2016), 30 were allocated to the intervention and 34 to the control arm (Figure 1)... Participants with complete data (n=61) were included in analyses of primary, secondary, adherence, and feasibility outcomes"		
**13b) CONSORT: For each group, losses and exclusions after randomisation, together with reasons**		
Figure 1		
**13b-i) Attrition diagram**		
NA		
**14a) CONSORT: Dates defining the periods of recruitment and follow-up**		
"We randomized 64 participants who were recruited over 5 months (July 2016- November 2016)...At the 8-week follow-up time (October 2016-January 2017), 3 out of 64 participants were unable to be contacted..."		
**14a-i) Indicate if critical "secular events" fell into the study period**		
NA		
**14b) CONSORT: Why the trial ended or was stopped (early)**		
NA		
**15) CONSORT: A table showing baseline demographic and clinical characteristics for each group**		
Table 1		
**15-i) Report demographics associated with digital divide issues**		
Did not collect this data		
**16a) CONSORT: For each group, number of participants (denominator) included in each analysis and whether the analysis was by original assigned groups**		
**16-i) Report multiple "denominators" and provide definitions**		
Table 2		
**16-ii) Primary analysis should be intent-to-treat**		

""We used an intention-to-treat analysis. We analyzed all women who completed baseline and 8-week surveys (complete cases) according to their randomization status."		
**17a) CONSORT: For each primary and secondary outcome, results for each group, and the estimated effect size and its precision (such as 95% confidence interval)**		
Table 2		
**17a-i) Presentation of process outcomes such as metrics of use and intensity of use**		
"Women in the intervention arm (n=30) attended 2.8 group video sessions per week on average for over 8 weeks. The attendance had a standard deviation of 1.17 and a skewed distribution with a median of 3.5. Participants attended 3.3 sessions per week in the rst half of the study and 2.4 sessions per week in the second half."		
**17b) CONSORT: For binary outcomes, presentation of both absolute and relative effect sizes is recommended**		
NA		
**18) CONSORT: Results of any other analyses performed, including subgroup analyses and adjusted analyses, distinguishing pre-specified from exploratory**		
Multimedia appendix 4.		
**18-i) Subgroup analysis of comparing only users**		
NA		
**19) CONSORT: All important harms or unintended effects in each group**		
NA		
**19-i) Include privacy breaches, technical problems**		
NA		
**19-ii) Include qualitative feedback from participants or observations from staff/researchers**		
Textbox 1		
DISCUSSION		
**20) CONSORT: Trial limitations, addressing sources of potential bias, imprecision, multiplicity of analyses**		
**20-i) Typical limitations in ehealth trials**		
"We relied on a self-report measure of physical activity, which though validated and widely used, could have introduced bias. Participants and investigators were not blinded to their randomization status, which could have also introduced bias. Our sample size limited our ability to fully explore the differences in outcomes by baseline activity status. In post hoc sensitivity analyses, we found that our model was neither sensitive to the removal of exercise time slot nor to 2 imputation strategies to address missing data. We were unable to test a longer intervention due to limited resources, and we could not assess whether the intervention effect could be maintained over a longer time due to our waitlist design. In addition, we were unable to fully disentangle the impact of videoconferencing separate from the impact of mobile apps. "		
**21) CONSORT: Generalisability (external validity, applicability) of the trial findings**		
**21-i) Generalizability to other populations**		
"Our digital tools helped create an efficient recruiting process; however, our recruitment and enrollment strategies and inclusion criteria resulted in a sample that was not representative of the United States population."		
**21-ii) Discuss if there were elements in the RCT that would be different in a routine application setting**		
NA		
**22) CONSORT: Interpretation consistent with results, balancing benefits and harms, and considering other relevant evidence**		
**22-i) Restate study questions and summarize the answers suggested by the data, starting with primary outcomes and process outcomes (use)**		
"This study suggests that technology can be used to create an individualized physical activity intervention with social support using a scalable and cost-effective delivery mechanism for mothers."		
**22-ii) Highlight unanswered new questions, suggest future research**		
"Further studies are needed to better establish how long these changes in physical activity can be maintained and whether these ndings can be reproduced in a more diverse population."		
Other information		
**23) CONSORT: Registration number and name of trial registry**		
"Trial Registration: ClinicalTrials.gov NCT02805140; https://clinicaltrials.gov/ct2/show/NCT02805140 (Archived by WebCite at http://www.webcitation.org/6yYZwRveg)"		
**24) CONSORT: Where the full trial protocol can be accessed, if available**		

Protocol description included in methods.		
**25) CONSORT: Sources of funding and other support (such as supply of drugs), role of funders**		
"MNM was supported by a National Institutes of Health (NIH) grant T32AT003997 from the National Center for Complementary and Integrative Health (NCCIH). FH was supported by NIH grant K24 AT007827 from NCCIH."		
**X26-i) Comment on ethics committee approval**		
"Before recruitment, we received approval from the University of California, San Francisco Institutional Review Board (14-15344), and registered our trial with the Clinical Trials Registry (NCT02805140)."		
**x26-ii) Outline informed consent procedures**		
"Once on the study website, women were able to sign up for an introductory phone call in which study staff reviewed the study procedures and reviewed consent forms using Docusign before enrollment began."<br>"Informed consent was obtained by study staff in the introductory phone call and consent forms were electronically signed during or after the call."		
**X26-iii) Safety and security procedures**		
NA In their consent forms, participants were asked to contact primary investigator in the event of an adverse event or the IRB, however this was considered a low risk study and so we did not include these details in the paper.		
**X27-i) State the relation of the study team towards the system being evaluated**		
"None declared."		
