# Supplementary figures and images for: Increasing Physical Activity in Mothers Using Video Exercise Groups and Exercise Mobile Apps: Randomized Controlled Trial
Source: J Med Internet Res. 2018 May 18;20(5):e179. doi: 10.2196/jmir.9310 (PMC5984277; doi:10.2196/jmir.9310)

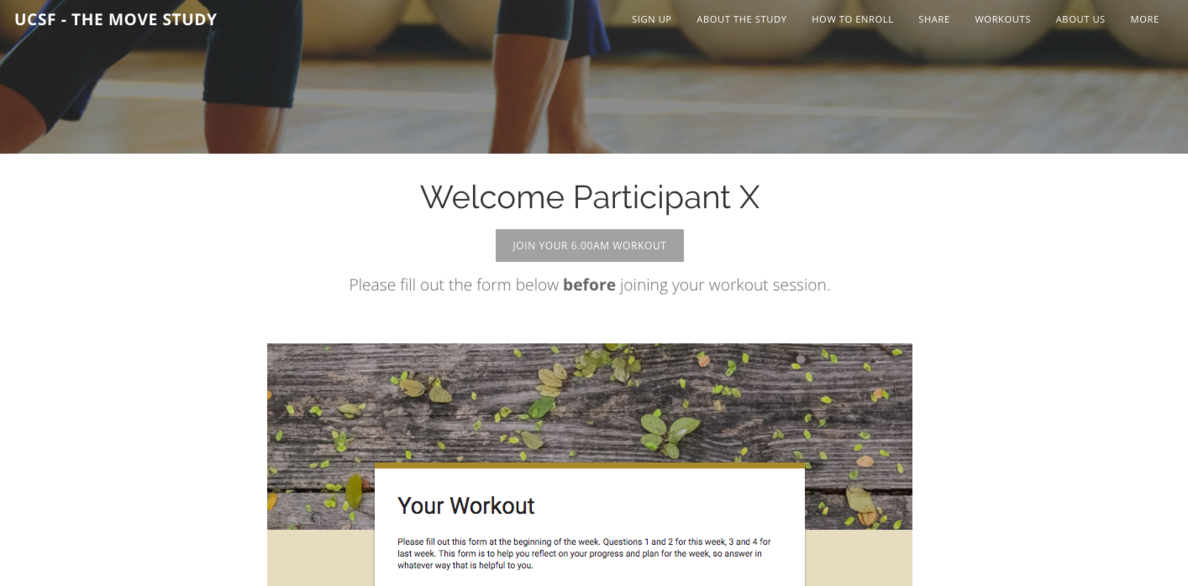

Supplement: Multimedia Appendix 2 [file jmir_v20i5e179_app2.png]
