# Supplementary material for: Increasing Physical Activity in Mothers Using Video Exercise Groups and Exercise Mobile Apps: Randomized Controlled Trial
Source: J Med Internet Res. 2018 May 18;20(5):e179. doi: 10.2196/jmir.9310 (PMC5984277; doi:10.2196/jmir.9310)
Supplement: Multimedia Appendix 3 [file jmir_v20i5e179_app3.pdf]

Multimedia Appendix 3

Recommended exercise apps and websites

Apps
7M Workout - Johnson & Johnson - https://7minuteworkout.jnj.com/
Nike+ Training - https://www.nike.com/us/en_us/c/nike-plus/training-app
Sworkit - https://sworkit.com/
**YouTube videos**
Cardio & Strength
Fitness Blender: Fat Burning HIIT Cardio Workout - https://www.youtube.com/watch?v=VhdXXqcoco0
Yoga & Pilates
Beginner Morning Yoga Sequence for Greatist - https://www.youtube.com/watch?v=lJi17P7iKNM
Beginner Yoga Flow for Greatist - https://www.youtube.com/watch?v=WXiiczhzox4
Fitness Blender: Leg Slimming Pilates Butt and Thigh Workout - https://www.youtube.com/watch?v=Womx4TM6p3A
POP Pilates for Beginners - https://www.youtube.com/watch?v=lCg_gh_fppI
Power Yoga for Beginners - https://www.youtube.com/watch?v=MFMMOEUGjeA
Dance
8 Counts - Zumba - https://www.youtube.com/watch?v=g27wcIl0lko
Dance Fitness with Jessica - Watch me - https://www.youtube.com/watch?v=lsqchmRovB8
Dance Fitness with Jessica - Worth it - https://www.youtube.com/watch?v=r04vFxBqRK8
Fitness Marshall - Can't Stop the Feeling - https://www.youtube.com/watch?v=M5ffOxDIcwc
Fitness Marshall - Cheap Thrills - https://www.youtube.com/watch?v=iL6ufJLkiUo
Fitness Marshall - Sorry - https://www.youtube.com/watch?v=sSkJZNY1D3Q
Nilesh Fatnani - Bollywood/Zumba workout - https://www.youtube.com/watch?v=Ic-RuGRgaxE
Yahsuh Dance Fitness - Boom, Boom - https://www.youtube.com/watch?v=RtZop9VA9Uc
Yahsuh Dance Fitness - I'm into you- https://www.youtube.com/watch?v=3YxoCj_hfiY
