# Supplementary material for: Increasing Physical Activity in Mothers Using Video Exercise Groups and Exercise Mobile Apps: Randomized Controlled Trial
Source: J Med Internet Res. 2018 May 18;20(5):e179. doi: 10.2196/jmir.9310 (PMC5984277; doi:10.2196/jmir.9310)
Supplement: Multimedia Appendix 4 [file jmir_v20i5e179_app4.pdf]

Multimedia Appendix 4

Table 4. Post hoc Sensitivity Analyses - differences across randomization arms in physical activity changes over 8 weeks for inactive mothers and all mothers

	Inactive Mothers			All Mothers		
Models	MVPA[a]	Vigorous	Moderate	MVPA[a]	Vigorous	Moderate
**Original Model[b]**						
Treatment Effect	50.0	19.0	9.7	42.2	13.2	13.8
P value	.03	.02	.36	.12	.20	.14
**Model without time variable[c]**						
Treatment Effect	50.3	18.5	11.0	41.5	11.9	15.6
P value	.03	.03	.27	.11	.25	.09
**Model with total children variable[d]**						
Treatment Effect	41.4	16.1	8.3	41.8	13.7	12.5
P value	.06	.04	.42	.12	.19	.18
**Model imputing missings assuming no change[e]**						
Treatment Effect	51.3	17.9	14.4	41.5	12.4	16.9
P value	.02	.02	.17	.11	.20	.07
**Model imputing missings assuming worst case[f]**						
Treatment Effect	44.2	15.5	9.7	33.3	9.1	12.6
P value	.049	.047	.34	.20	.36	.17

[a]MVPA: moderate to vigorous physical activity

[b]Original model, adjusted for outcome value at baseline and exercise time slot, for inactive (n=51) and all (n=61) mothers

[c]Model with no time variable, adjusted for outcome value at baseline for inactive (n=51) and all (n=61) mothers

[d]Model with 'total children' variable added to model, adjusted for baseline value of outcome and exercise time slot, for inactive (n=51) and all (n=61) mothers

[e]Model imputing missing values assuming no change where all missing 8 week estimates (n=3) are equal to baseline estimates, adjusted for outcome value at baseline and exercise time slot for inactive (n=54) and all (n=64) mothers

[f]Model assuming worst case where all missing values for intervention (n=1) are equal to mean difference in the intervention arm minus 1 standard deviation and missing values for control (n=2) are equal to mean difference in control arm plus 1 standard deviation, adjusted for outcome value at baseline and exercise time slot for inactive (n=54) and all (n=64) mothers
